# Profiles of Dysarthria: Clinical Assessment and Treatment

**DOI:** 10.3390/brainsci14010011

**Published:** 2023-12-22

**Authors:** Wolfram Ziegler, Anja Staiger, Theresa Schölderle

**Affiliations:** Clinical Neuropsychology Research Group (EKN), Institute of Phonetics and Speech Processing, Ludwig-Maximilians-University, 80799 Munich, Germany; anja.staiger@ekn-muenchen.de (A.S.); theresa.schoelderle@ekn-muenchen.de (T.S.)

In recent decades, we have witnessed a wealth of theoretical work and proof-of-principle studies on dysarthria, including descriptions and classifications of dysarthric speech patterns, new and refined assessment methods, and innovative experimental intervention trials. Thanks to this research, immense clinical knowledge has been accumulated, and powerful methods have been developed, which are waiting to be made applicable for speech–language therapists and caregivers in their daily clinical work.

This Special Issue aims to explore where we are today in terms of translating this knowledge into standard clinical practice.

- Back in the 1960s, Frederic Darley’s group provided us with a rich inventory of *auditory perceptual* descriptors of dysarthric speech [1], but since then, little work has been carried out to compensate for the psychometric downsides of this approach and implement it as a reliable standard in clinical assessment [2]. Questions regarding the grain size of reliably assessable auditory perceptual dimensions of dysarthric speech, the design of rating protocols that support the consistency of perceptual judgments, or the number of ratings per diagnostic parameter that may grant statistically robust results must be answered to improve the psychometric properties of the “gold-standard” method of dysarthria assessment [3]. Some of the auditory perceptual dimensions discussed in the literature, e.g., *intelligibility* or *naturalness*, are particularly informative in terms of the communicative resources a patient uses in their communicative activities [4]. As the conditions of clinical diagnostics pose particular challenges to a reliable and valid assessment of such parameters, research is needed to facilitate their implementation as a regular part of standard dysarthria profiling [5].
- Since the 1930s, when the first oscillograms displaying *acoustic features* of speech impairments in stroke patients were published [6], a vast number of papers presenting acoustic patterns of dysarthria have appeared. Over the decades, the whole repertoire of speech signal parameters has been applied to objectify and quantify dysarthric impairment, including plosive voice onset times, vowel formants, fricative spectral parameters, voice fundamental frequency, or measures of rhythm and timing [7]. Yet, despite continuous automatization efforts [8], it has still not been sufficiently clear, until today, how the promise of diagnostic objectivity of acoustic dysarthria profiles can be fulfilled in daily clinical practice. Numerous problems remain unresolved, such as the specification of measures that are robust and clinically interpretable across the whole range of speech patterns seen in clinical practice, or the provision of representative standard norms for such measures.
- Likewise, increasingly sophisticated electronic devices have been introduced to study *speech movement characteristics* in persons with dysarthria; most importantly Electromagnetic Articulography (EMA; [9]) or, more recently, real-time MRT (e.g., [10]). New ways of visualizing a speaker’s tongue movements in near real time also fuel hopes of using such techniques in the development of effective biofeedback- or VR-based gaming interventions [11]. Yet, the “physiologic approach” based on such tools, as propagated by motor speech pioneers as early as the 1970s [12], is still a long way from becoming firmly established in clinical care, and existing studies are still based on small sample sizes [13]. Again, questions regarding the clinical validity of standard kinematic parameters, and the lack of representative standard norms that can capture the enormous intra- and inter-speaker variability in articulator movements, remain unanswered; not to mention the unsolved problem of how the high technical standards and the specific expertise required in operating such complex assessment tools can be implemented and maintained in clinical settings.
- As a recent technical development, the application of *machine learning* technology in dysarthria research has increased enormously, with a growth of about 900% since the beginning of this century. However, despite the great promise of this powerful technology, reports on standard applications in clinical care are still lacking and approaches based on methodologically rigorous models still yield disappointing results [14,15]. Among the major obstacles that need to be overcome is the problem of how we can collect training datasets that guarantee accurate and unbiased predictions, particularly considering the large variability that exists across dysarthria types, disease stages, degrees of severity, and individual idiosyncrasies; not to mention the variability as a function of age and gender, dialectal variants, ethnicities, mood, motivation, or factors related to the quality of acoustic data. The black-box nature of AI solutions bears the risk of unrecognized biases, which are potentially caused by these confounders, and thereby create unknown and potentially harmful limitations. Clinically safe, explainable, and therapeutically interpretable AI architectures are needed to help clinicians in therapeutic decision making [16].
- A fundamental issue that looms behind all diagnostic and therapeutic approaches concerns the status of *nonspeech* or *paraspeech* tasks or exercises and how they relate to speech [17]. Syllable repetition, vowel prolongation, silent lip or tongue movements, and other vocal tract maneuvers have a firm place in most dysarthria assessment protocols and treatment approaches [18]; although their validity as measures of speech impairment has been challenged theoretically [19] and has rarely been tested empirically [20].
- Considering that the ultimate goal of all clinical and technological advancements is to help persons with dysarthria manage their everyday life, clinical research must also develop and refine robust ways of measuring *communicative participation* and constantly test the available diagnostic parameters for their predictive validity in relation to participation success [21]. The development of *patient reported outcome measures* (PROMs) as indicators of participation, and of communication-related speech parameters which serve as potential predictors, will becom increasingly important in this research [22].

The defining objective of translational research is to translate and disseminate the advancements made in these fields into real-world practice, i.e., for the provision of healthcare, and to identify the gaps and limitations that exist [23]. Clinical research must be committed to continuously monitor its progress towards this goal. With this in mind, this Special Issue, entitled *Profiles of Dysarthria: Clinical Assessment and Treatment,* intends to draw a picture of the current state of translating dysarthria research into clinical care. Nine manuscripts, submitted by leading research groups from the US, Canada, and Europe, were accepted for publication following a rigorous review process. The contributions listed below place a spotlight on some of the most topical issues within current clinical research.

The first two articles in the List of Contributions deal with the gold standard method of dysarthria assessment, i.e., the auditory perceptual evaluation of the dimensions and typologies within dysarthric speech.

**Fougeron et al.** report on new psychometric evaluations of *MonPaGe*, a standardized clinical assessment tool based on auditory perceptual and acoustic analyses of dysarthric speech in French speaking countries. Complementing previous evidence of the reliability and validity of the MonPaGe protocol, classifiers were developed in this study to allow differentiation between various types of motor speech disorders.

**Kim and colleagues** address the dysarthria classification problem from a different perspective. In their experiment they used auditory *free classification* to detect dysarthria subtypes within a group of patients with Huntington’s disease; a method in which expert listeners arrange patients’ speech samples according to their perceived acoustic similarity. The authors showed that comparing free classification subgroups with subgroups obtained via conventional statistical clustering can help identify speech characteristics that are particularly relevant to differential diagnosis.

Four articles are concerned with questions regarding the relationship between nonspeech vocal tract motor control and dysarthria. **G. Weismer’s** position paper gives an introduction and an exhaustive and critical overview of the comprehensive literature that already exists on this topic. One of the conclusions of this article is that nonverbal oral motor tasks continue to be surprisingly popular in the clinical assessment of motor speech disorders, despite the fact that practically no empirical and theoretical evidence is available to support the use of this method.

The study by **Clark et al.** was focused on a prominent nonspeech paradigm within dysarthria research, i.e., the objective assessment of orofacial weakness. The primary research question in this article is whether the maximum strengths of the lips and tongue vary across different types of dysarthria. Confirming their expectations, patients with paretic motor syndromes, unlike those with ataxia, had reduced maximum orofacial strength, whereas a group including mostly Parkinson’s syndrome patients showed less straightforward results.

**Ziegler et al.** tested two hypotheses that are central to the use of nonspeech parameters in the assessment of speech impairment [17], i.e., that speech and nonspeech diagnostic parameters split along effector (lip vs. tongue) or functional (speed vs. accuracy) boundaries rather than along the boundary separating the verbal from the nonverbal oral motor domain. The findings of this study failed to support these hypotheses and contradicted the view that nonspeech parameters could be useful in delineating the speech characteristics of persons with dysarthria.

**Kuruvilla-Dugdale and Mefferd** counter the argument that nonspeech tasks are indispensable for the systematic control of articulatory demands in clinical assessment. They used 3D EMA to develop natural, single-word materials that are carefully controlled for kinematic parameters such as the movement of several articulators, and then applied these materials to the study of persons with dysarthria to identify demand- and disease-specific articulatory performance characteristics in these patients.

Two further papers focus on one of the most extensively studied communication-related speech parameters, i.e., *intelligibility*. **Hirsch et al.** investigated the reliability and validity of intelligibility ratings for adult speakers with dysarthria. Their particular interest was in validating the ratings provided by speech–language pathologists by comparing them with orthographic transcripts. In a similar vein, **Soriano et al.** addressed methodological issues affecting intelligibility assessment in children with cerebral palsy by comparing parents’ ratings of their children’s intelligibility with transcription scores. Both studies support the use of subjective estimates of intelligibility in clinical practice, but with the limitation that the validity and reliability of intelligibility ratings vary considerably depending on the raters’ experience and familiarity with the speakers they are diagnosing.

The last paper in the List of Contributions addresses the question of how the impact of speech impairment can be assessed at the *participation level*, i.e., at the level of the patients’ everyday communication experience. **Page and Yorkston** contributed a topical position paper on this issue. After defining and describing the complexity of participation-oriented research, they discuss the still unresolved problem of how an impaired person’s success in everyday communication can be predicted by data collected in clinical testing. The authors conclude that communicative participation should be a primary focus of treatment planning and intervention, and that specifically developed diagnostic tools are required for this purpose.

## Data Availability

Not applicable.
